# Ischemic stroke outcome prediction with diversity features from whole brain tissue using deep learning network

**DOI:** 10.3389/fneur.2024.1394879

**Published:** 2024-05-03

**Authors:** Yingjian Yang, Yingwei Guo

**Affiliations:** ^1^School of Electrical and Information Engineering, Northeast Petroleum University, Daqing, China; ^2^Shenzhen Lanmage Medical Technology Co., Ltd, Shenzhen, Guangdong, China

**Keywords:** acute ischemic stroke, outcome prediction, whole brain, deep learning, machine learning

## Abstract

**Objectives:**

This study proposed an outcome prediction method to improve the accuracy and efficacy of ischemic stroke outcome prediction based on the diversity of whole brain features, without using basic information about patients and image features in lesions.

**Design:**

In this study, we directly extracted dynamic radiomics features (DRFs) from dynamic susceptibility contrast perfusion-weighted imaging (DSC-PWI) and further extracted static radiomics features (SRFs) and static encoding features (SEFs) from the minimum intensity projection (MinIP) map, which was generated from the time dimension of DSC-PWI images. After selecting whole brain features F_fuse_ from the combinations of DRFs, SRFs, and SEFs by the Lasso algorithm, various machine and deep learning models were used to evaluate the role of F_fuse_ in predicting stroke outcomes.

**Results:**

The experimental results show that the feature F_fuse_ generated from DRFs, SRFs, and SEFs (Resnet 18) outperformed other single and combination features and achieved the best mean score of 0.971 both on machine learning models and deep learning models and the 95% CI were (0.703, 0.877) and (0.92, 0.983), respectively. Besides, the deep learning models generally performed better than the machine learning models.

**Conclusion:**

The method used in our study can achieve an accurate assessment of stroke outcomes without segmentation of ischemic lesions, which is of great significance for rapid, efficient, and accurate clinical stroke treatment.

## 1 Introduction

Worldwide, ~13.7 million people endure stroke annually, leading to ~5.8 million deaths, while approximately one-third of survivors will be present with varying degrees of disability (1, 2). Acute ischemic stroke is the primary type of stroke, with a prevalence ratio of 85–90% (3). With the continuous progress of medical imaging methods and analysis technology, the mortality rate of stroke has been reduced, but the disability rate has not been effectively improved (4). Assessing the rehabilitation ability of patients in advance would be beneficial for devising clinical therapy plans, disease detection, and therapeutic management in the treatment of stroke disease (5).

In most well-developed countries, stroke outcomes have been greatly improved. These advancements have been achieved depending on the highly effective recanalizing therapies, high-resolution imaging technology, and standardized care for patients across medical departments (6). With the evolution of medical technology, significant progress has been achieved in outcome prediction (7–11). However, most of these methods require clinical physicians to collect basic information about patients, evaluate their status, and score them beforehand, wherein the National Institutes of Health Stroke Scale (NIHSS) has become a widely used indicator of stroke severity (12), and modified Rankin Scale (mRS) has been used as the most common score to evaluate patient outcomes (13). Most clinical scores have the same common limitations in that their evaluation usually depends on clinicians' judgment of the patient's expression, but the subjective expressions and judgments may influence treatment planning and patient prognosis. Although clinical scores and basic patient information have been proven to express prognosis to a certain extent, it is difficult to improve when using them to predict stroke outcomes significantly.

As medical imaging provides more intuitive and abundant image data, research studies are gradually proposed based on clinical scores, basic information about patients, and medical images. Various studies (14–18) explored the role of medical images in predicting rehabilitation levels, but the overall performance failed to outperform the non-imaging data. With the advent of radiomics technology, the detailed quantification of medical images has been achieved, promoting an in-depth analysis of medical imaging in disease diagnosis, treatment, and prevention (19–23). Inspired by this technology, the radiomics features calculated from the diseased regions were used in the outcome prediction task, but it is challenging to outperform the non-imaging data (14, 24–26). Then, the combination of non-imaging data and radiomics features was proposed. Zhou et al. (27) proposed a prediction model based on essential clinical information of patients and radiomics features calculated from the diffusion-weighted imaging (DWI) and apparent diffusion coefficient (ADC) maps, outperforming the models based on individual, clinical, or radiomics features. Quan et al. (28) proved that the predicting ability of the model would be improved after introducing the radiomics feature. It can be seen that the fusion of clinical information improved the prediction of patients' recovery levels and the emergence of new technologies.

Different from the above feature analysis of three-dimensional (3D) images, studies (29, 30) found that the novel dynamic radiomics features (DRFs) extracted from dynamic susceptibility contrast perfusion-weighted imaging (DSC-PWI) can characterize blood flow transmission status and effectively enhance the expression ability of medical images in outcome prediction tasks by combining with clinical text information. Furthermore, a prediction model (31) using the combined features of ischemic lesions and whole brain tissue was proposed and achieved similar accuracy to the models based on non-imaging data and lesion tissue. However, timely treatment for stroke was an essential condition to improve the rehabilitation of patients (32). The basic information about patients and lesion characteristics used in the above-mentioned prognostic studies require extra time for consultation and lesion segmentation, reducing the efficiency of clinical treatment.

Previous works to predict stroke outcomes can be divided into classical machine learning methods (7–11) and deep learning-based methods (10, 14). It is comprehendible that training a deep learning model requires a large amount of clinical data. Due to the privacy protection policy and limited labeled data in the healthcare unit, it is challenging to collect abundant samples. Transfer learning was proposed to solve this issue in small data scenarios (33). Then, some studies used the transfer learning theory to diagnose stroke (34, 35), stroke risk prediction (36), and stroke outcome prediction (37–39). Among present pre-trained models, Med3D, which is pre-trained by eight different datasets, was regarded as a trusted feature extractor in multiple medical scenarios (40–42). However, most of these studies only took medical images or physiological data as the input data, lacking complete information, and it has not been carried out in stroke prognosis.

Based on the above, this study proposed a stroke outcome prediction method based on the combined strategy of dynamic and static features extracted from the whole brain. The features in multiple dimensions and states were calculated through in-depth mining of features in the whole brain, and the prediction accuracy was improved. The significant contributions and innovations of this study are listed below.

a) This study proposed a rapid and efficient stroke outcome prediction model. According to this model, segmenting ischemic lesions or presenting basic patient information is unnecessary. The method used in our study is only dependent on the image features extracted from the brain parenchyma to predict the recovery level of patients, thus saving time for segmentation and consultation.

b) This study used diversity methods to extract the whole brain features, including DRFs obtained from DSC-PWI images, the static radiomics features (SRFs) extracted from the minimum intensity projection (MinIP) map of DSC-PWI images in time direction, and static encoding features (SEFs) extracted from the MinIP map by the pre-trained Med3D model. The complete and comprehensive feature extraction from the whole brain provides more research basis for clinical practice.

c) This study compared the performance of the deep learning and machine learning models in predicting ischemic stroke outcomes, demonstrating the advantages of the deep learning models. In addition, due to the introduction of the pre-training model, the requirement for datasets is reduced.

## 2 Materials and methods

### 2.1 Materials

The datasets used in this study were collected from Shanghai Fourth People's Hospital Affiliated with Tongji University School of Medicine and exempted from informed consent. The datasets were collected from 2013 to 2016 and included 161 DSC-PWI images collected from 88 hospitalized patients.

The inclusion criteria for this study are as follows: (1) all patients were adults and scanned for DSC-PWI and DWI images within 24 h of admission and (2) the patients lacking basic or imaging information were removed from the queue. Finally, 156 DSC-PWI images were selected to be used in our study. The DSC-PWI images in our datasets were collected on a 1.5-T Avanto scanner (Siemens, Erlangen, Germany). The imaging parameters were listed as follows: Slices = 19, TH = 5 mm, TR = 1,520 ms, TE = 32 ms, measurements = 50, FOV = 230 mm^2^, matrix size = 128 × 128, and band width = 1,346 Hz/pixel. The gadopentetate dimeglumine (Gd-DTPA) (Shanghai Pharmaceutical Corporation, Shanghai, China) was injected at a dose of 0.2 mmol/kg body weight and a saline flush of 30 ml at the same injection flow rate of 4 ml/s.

Among the datasets, the ratio of male to female samples was 25%, and the mean ± variance of age, income NIHSS score, outcome NIHSS, modified Rankin scale (mRS) score within 90 days, and the onset time were 69.67 ± 11.02, 8.62 ± 6.69, 4.07 ± 5.29, 1.91 ± 2.13, and 7.05 ± 10, respectively. The mRS score is widely used to evaluate the disability of stroke patients. Regarding patients' basic information, the ratio of patients with left and right limbs weakness, lisp out, confusion, hypertension, diabetes, and atrial fibrillation were 40.9, 43.2, 65.9, 11.4, 67, 29.5, and 25%. The basic statistics for datasets used in this study are shown in Table 1.

**Table 1:** The basic information of datasets in our study.

**Items**	**Value**
Patients number	88
Volumes of datasets	156
Female (%)	39 (44.3%)
Age (mean ± Std)	69.67 ± 11.02
Income NIHSS score (mean ± Std)	8.62 ± 6.69
Outcome NIHSS score (mean ± Std)	4.07 ± 5.29
90-day mRS	1.91 ± 2.13
Onset time (hour)	7.05 ± 10
Patients with left limb weakness (%)	36 (40.9%)
Patients with right limb weakness (%)	38 (43.2%)
Patients with lisp out (%)	58 (65.9%)
Patients with confused (%)	10 (11.4)
Patients with hypertension (%)	59 (67%)
Patients with diabetes (%)	26 (29.5%)
Patients with atrial fibrillation	22 (25%)

### 2.2 Methods

**Figure 1:**
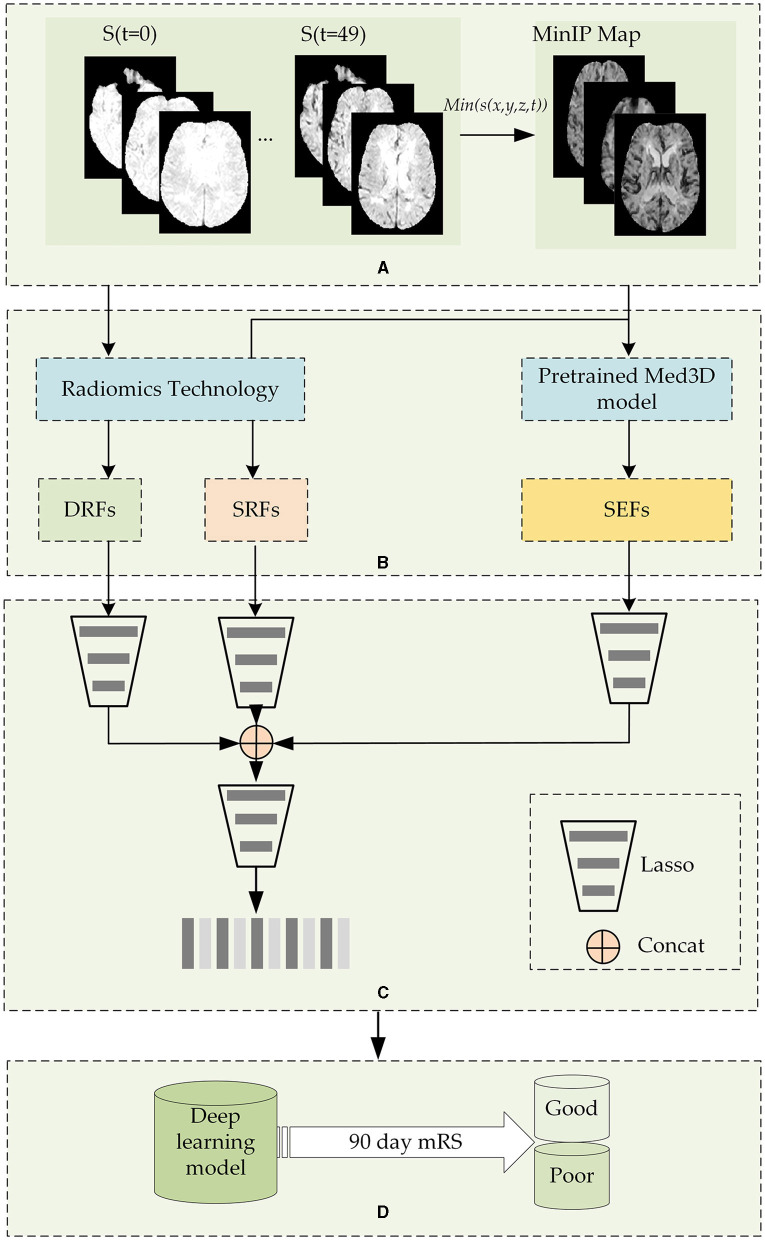
The flowchart of the stroke outcome prediction. **(A)** Constructing the MinIP map on the time dimension of DSC-PWI images; **(B)** multidimensional whole brain features quantification; **(C)** feature dimension reduction; **(D)** stoke outcome prediction using deep learning models.

Figure 1 shows the flowchart of the stroke outcome prediction proposed in this study. As shown in Figure 1, the stroke outcome prediction method mainly includes (a) constructing the MinIP map on the time dimension of DSC-PWI images, (b) multidimensional whole brain features quantification, (c) feature dimension reduction, and (d) stroke outcome prediction.

#### 2.2.1 Construction of the MinIP map on the time dimension of DSC-PWI images

DSC-PWI image comprises 50 three-dimensional (3D) images scanned in continuous moments, and the time-intensity curve of each voxel in the DSC-PWI image represents the change in gray level before, during, and after the arrival of the contrast agent. Notably, when the contrast agent arrives, the gray level of abnormal ischemic tissue exhibits a lesser and slower decrease than the normal tissue and may even remain unchanged (43, 44). Therefore, the minimum gray level of voxels in the time dimension provides the blood flow condition of brain tissue. Based on this insight, this study performed MinIP processing of the DSC-PWI image on the time dimension and obtained the MinIP map.

Before the MinIP processing, the preprocessing of datasets should be performed to remove position deviation. In this step, the rigid registration of 3D images in the time dimension of the DSC-PWI image was followed by the simple-elastix package in Python (45). Then, the software FSL (46) was used to segment the skull and brain tissue regions.

After image preprocessing, the MinIP map of the voxels in the brain tissue was obtained by executing the MinIP processing on the time dimension of the DSC-PWI image. The expression to obtain the MinIP map is shown in Equation (1). In this way, the blood flow transmission ability of different tissues in the DSC-PWI image can be preserved, and the image was reduced from four dimensions to three dimensions, saving the operation cost of subsequent work and improving the calculation speed.


(1) $$\begin{array}{l} MinIP\;\left(x,y,z\right)\;=\text{min }intensity\;\left(x,y,z,:\right) \end{array}$$

Items *x, y*, and *z* represent the coordinate values in three-dimensional space, respectively, and the minimum function *min()* was used to obtain the minimum value in the time-intensity sequence of (x, y, z) of the DSC-PWI image.

#### 2.2.2 The quantification of multidimensional whole brain features quantification

The DSC-PWI image and the MinIP map reflect the different aspects of the blood flow transmission of brain tissue. The DSC-PWI image shows the gray level changes in brain tissue at different times and then shows the dynamic transmission process of cerebral blood flow. The MinIP map represents the response of different brain tissues to the contrast agent. Through the complete feature extraction of DSC-PWI and the MinIP map, brain tissue's current state and recovery ability may be fully characterized.

This study used diverse methods to quantify the whole brain features and achieve the multidimensional quantification of dynamic and static features. For the dynamic features, this study used radiomics technology to extract the whole brain dynamic radiomics features (DRFs) from DSC-PWI images. For the static features, this study used radiomics technology and the pre-trained model Med3D to calculate the whole brain static features from the MinIP maps.

(a) Extracting DRFs of the whole brain

**Figure 2:**
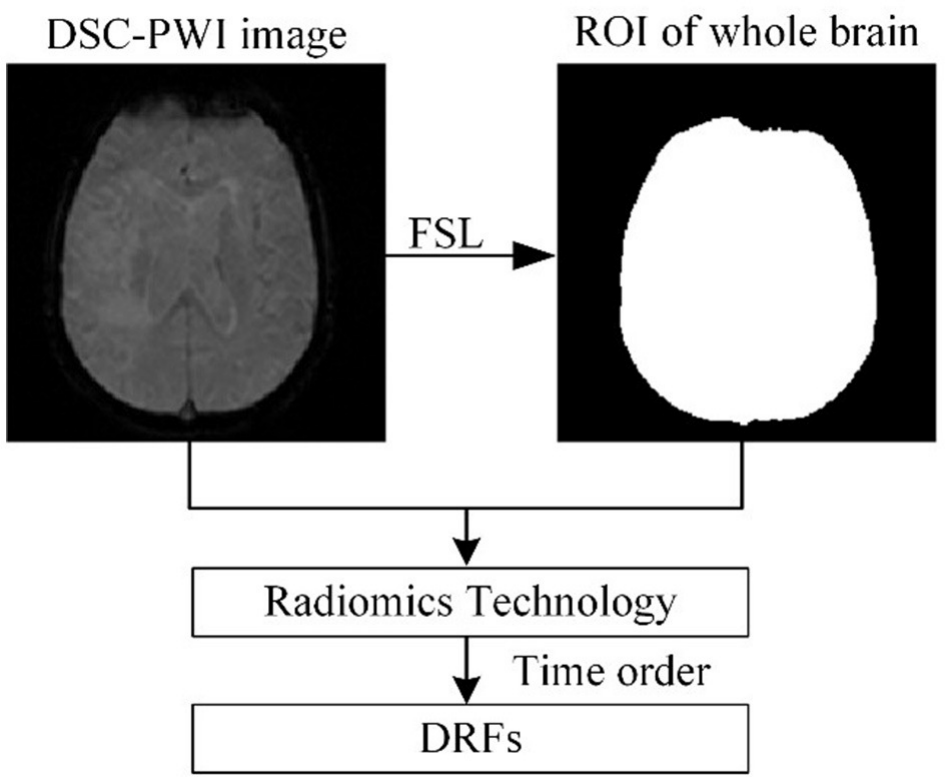
The process of extracting DRFs.

Figure 2 shows the process of extracting DRFs. First, the brain tissue region can be segmented based on the preprocessing step mentioned above. Then, this study divided the DSC-PWI image into *N* (*N* = 50) 3D brain images and calculated the radiomics features of the whole brain region in each 3D image. The feature calculation was implemented with the PyRadiomics package in Python. Then, the DRFs were obtained by combining the radiomics features of each 3D image according to the time order.

When calculating the radiomics features, six feature groups can be found in the DRFs, including First-Order Statistics (First-order), Gray-Level Co-Occurrence Matrix (GLCM), Gray-Level Run-Length Matrix (GLRLM), Gray-Level Dependence Matrix (GLDM), Gray-Level Size-Zone Matrix (GLSZM), and Gand Neighboring Gray-Tone Difference Matrix (NGTDM). In detail, the DRFs had 84,400 features, including First-Order features, GLCM features, GLRLM features, GLSZM features, NGTDM features, and GLDM features.

(b) Static encoding features of the whole brain

**Figure 3:**
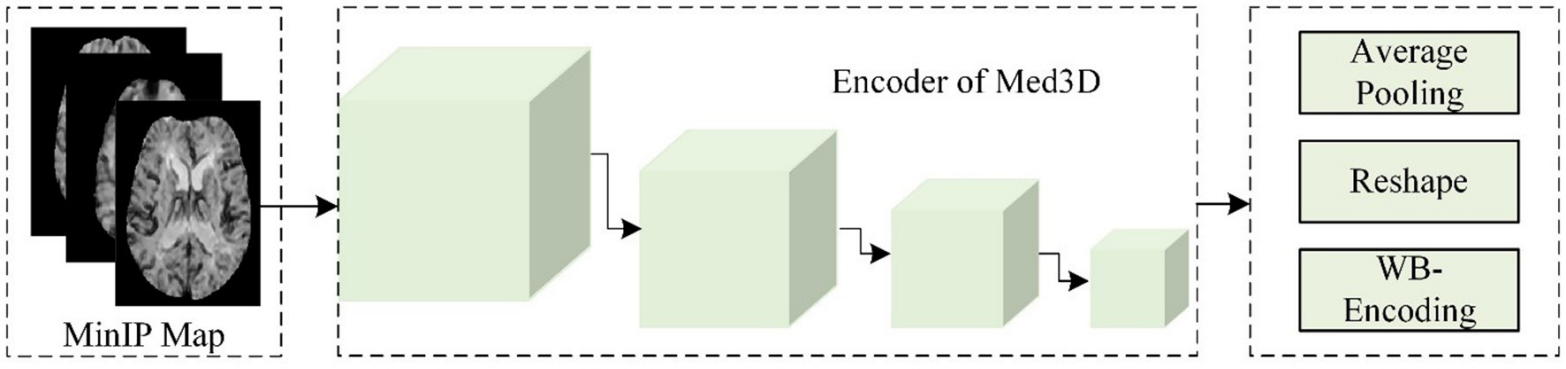
The flow chart of extracting SEFs.

As shown in Figure 3, this study used the Med3D model to analyze the MinIP image and calculated the static encoding features (SEFs) of whole brain tissue. In detail, this study used the MinIP map as the input of the encoder of Med3D to extract the SEFs. The output of the last layer of the encoder was performed with average pooling and feature expansion, and then one-dimensional features were obtained. Considering that the gray value of voxels in the MinIP map was the response to the arrival of contrast agents on brain tissue, the extracted SEFs can be used to evaluate the state of blood flow propagation.

(c) Static radiomics features of the whole brain

To extract the whole brain static feature information more comprehensively, this study further applied radiomics technology to extract the radiomics features of the whole brain in the MinIP map. In contrast to the DRFs obtained based on DSC-PWI images directly, the whole brain static radiomics features (SRFs) can only provide information when the gray level of all the voxels was the lowest, thus it only included the radiomics feature information of one 3D MinIP map. Notably, the SRFs had a total of 1,688 features, including First-order features, GLCM features, GLRLM features, GLSZM features, NGTDM features, and GLDM features.

#### 2.2.3 Feature dimension reduction and combination

(a) Feature dimension reduction

Since the dynamic and static features (DRFs, SRFs, and SEFs) extracted from the whole brain include excessive redundant information, an effective feature dimensionality reduction is necessary to find the most relevant features (47). This study used multilevel feature dimension reduction methods to compress the original features, including the significant feature selection and outstanding feature selection. Before the reduction of feature dimension, the samples should be divided into patients with good outcomes (mRS ≤ 2) and poor outcomes (mRS > 2). Among the 156 samples, 65 samples showed poor outcomes while 91 showed good outcomes.

In the process of feature dimension reduction, the first step was normalizing the datasets with mean-variance normalization (48), which was used to eliminate the influence of dimension and value-range differences between features. Then, the *t*-test algorithm and the absolute shrinkage and selection operator (Lasso) method were executed for feature dimension reduction (49). Both these methods can compass the original features. Depending on the previous study (50) in which multiple feature selection methods were used and compared, it can be found that features extracted from the Lasso algorithm could accurately reflect cerebral blood flow changes and improve the classification ability of models. Based on this reason, this study applied the Lasso algorithm to extract efficient features.

In detail, the *t*-test algorithm was used to extract the significant features, namely, significant DRFs, significant SEFs, and significant SRFs. The Lasso algorithm selected the critical features with non-zero coefficients from significant features. We defined the selected features as DRFs, SRFs, and SEFs. The original features were named original DRFs, original SRFs, and original SEFs, respectively.

(b) Feature combination

In this step, the three features selected by the Lasso algorithm were concatenated. Since features from different images and methods may have redundancy, this study used the Lasso algorithm to optimize our study's combined features. Finally, this study obtained the combined features depending on the Equation (2).


(2) $$\begin{array}{l} F_{fuse}\;=\;LASSO\;\left(concat\;\left(DRFs,\;SRFs,SEFs\right)\right) \end{array}$$

where *concat* means concatenation of the three groups of selected features.

#### 2.2.4 Stroke outcome prediction

After obtaining the combination features F_fuse_ from original dynamic and static features (DRFs, SEFs, and SRFs), this study used four deep learning networks LSTM, CNN, RNN, and linear network (LN) to predict the stroke outcomes (90 days mRS). The input of the deep learning network was F_fuse_, and the output was the possibility of either a good outcome or a poor outcome. The training parameters of networks are shown in Table 2. To obtain a reliable result, a mean score (MS), calculated from five mean indexes, namely, mean area under the curve (mAuc), mean precision score (mPre), mean accuracy score (mAcc), mean F1 score (mF1), and mean Recall (mRecall), was used to evaluate the ability to predict stroke outcomes. The five indexes were obtained through a 10-fold cross-validation method, which was achieved using the StratifiedKFold function in the sklearn package. This function can ensure the balance of the ratio between positive and negative samples in the training set and the test set, improving the reliability of the prediction results. In detail, this study used the 10-fold cross-validation to evaluate the obtained features. We randomly and uniformly divide the data into 10 equal parts based on the labels before the experiment. Each iteration used one part as the test set and the remaining four parts as the training set. This process was repeated 10 times independently. For example, the data for one patient in the datasets would be used as the test set in one of the ten iterations and as the training set in the remaining nine iterations, and the parameters of the same model would be re-initialized and trained at each iteration independently of each other. This implies that the 10 iterations are independent, and the data for the same patient will not be used as a test sample and training sample at the same iteration. Using the 10-fold cross-validation, the bias of results due to patient differences can be reduced, and a reliable result can be obtained.

**Table 2:** The training parameters of deep learning networks.

**Items**	**Value**
Epoch	500
Batch size	20
Optimizer	Adam
Loss function	Cross entropy
Learning rate	0.01
Stop condition	The loss value was higher than the minimum loss 10 times

After performing the 10-fold cross-validation operation, ten groups of Auc scores, Acc scores, Pre scores, F1 scores, and Recall scores can be obtained from each prediction model, and the mean score, mAuc, mAcc, mPre, mF1, and mRecall can be calculated using Equation (3). The Acc, Pre, F1, and Recall were calculated using Equation (4), Auc was the area under the curve obtained by the sklearn.metrics package. The obtained mean scores were used to further calculate the MS score according to Equation (5). In Equation (4), TP and TN represent the number of positive samples predicted to be positive, and the number of negative samples predicted to be negative, while FP and FN represent the number of positive samples predicted to be negative, and the number of negative samples predicted to be positive. In Equation (3), the index represents the Auc, Acc, Pre, F1, and Recall, and k is the number of folds (10-fold in our study).


(3) $$\begin{array}{l} m\;\left(index\right)\;=\;\frac{1}{k}\overset{k}{\underset{i\;=\;0}{\sum }}\;index \end{array}$$

(4) $$\begin{array}{ll} ACC\; & =\;\frac{TP\;+\;TN}{TP\;+\;FP\;+\;TN\;+\;FN},\\ pre\; & =\;\frac{TP}{TP\;+\;FP},\\ Rcall\; & =\;\frac{TP}{TP\;+\;FN},\\ F1\; & =\;2*\;\frac{Pre*Recall}{Pre+Recall}. \end{array}$$

(5) $$\begin{array}{l} MS\;=\;\left(mAuc\;+mPre\;+mAcc\;+\;mF1\;+mRecall\right)/5 \end{array}$$

In our study, nine machine models were selected to make a competitive result. The machine learning models included support vector machine (SVM), decision tree (DT), AdaBoost classifier (Ada), random forest (RF), k-nearest neighbors (KNN), logistic regression (LR), linear discriminant analysis (DA), gradient boosting classifier (GBDT), and GaussianNB (NB).

Besides, to thoroughly verify the role of SEFs extracted from Med3D, this study conducted comparative experiments with four encoders, namely, ResNet10, ResNet18, ResNet34, and ResNet50, of the pre-trained Med3D model. Thus, a comparison between the encoder and feature dimensions can be made.

## 3 Results

### 3.1 The performance of F_*fuse*_

**Figure 4:**
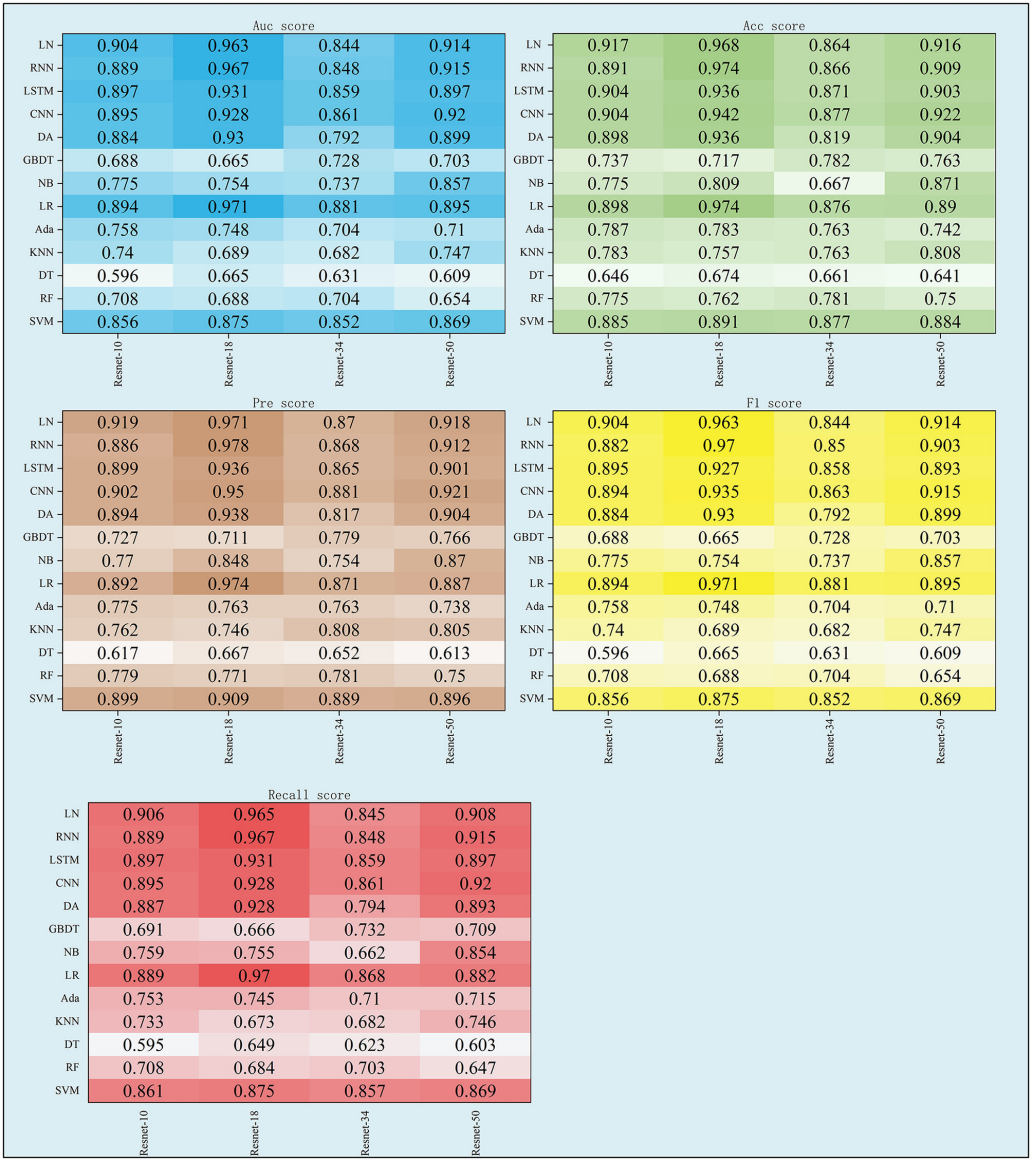
The scores of five indexes of F_fuse_ on the four DL models and nine ML models.

As shown in Table 3 and Figure 4, the performance of F_fuse_ on the DL models was better compared to the ML model. In detail, F_fuse_ based on Resnet-10 achieved the best MS scores of 0.893 and 0.91 on the ML models and the DL models, and the 95% confidence interval (95% CI) were (0.705, 0.849) and (0.884, 0.913), respectively. Besides, F_fuse_ based on Resnet 18 achieved the best performance in this experiment. The best MS score for both ML and DL groups was 0.971, and the 95% CI values were (0.703, 0.877) and (0.92, 0.983), respectively. For F_fuse_ based on Resnet 34, the best MS scores on the ML models and the DL models were 0.875 and 0.868. Although the best MS score of the ML model was better compared to the DL model, the overall MS scores of the DL models [CI 95% (0.849, 0.87)] were higher than that of the ML models [CI 95% (0.70, 0.817)]. For F_fuse_ based on Resnet 5, the best MS scores for ML and DL groups were 0.9 and 0.92, respectively, and the 95% CI for both groups were (0.706, 0.863) and (0.896, 0.925), respectively.

**Table 3:** The MS scores of F_fuse_ obtained from four encoders of Med3D on nine ML models and four deep learning models.

	**Models**	**Resnet10**	**Resnet18**	**Resnet34**	**Resnet50**
ML models	SVM	0.871	0.885	0.865	0.877
	RF	0.735	0.718	0.735	0.691
	DT	0.610	0.664	0.639	0.615
	KNN	0.751	0.711	0.724	0.771
	Ada	0.766	0.757	0.729	0.723
	LR	0.893	0.971	0.875	0.890
	NB	0.771	0.784	0.711	0.862
	GBDT	0.706	0.685	0.750	0.729
	DA	0.889	0.933	0.803	0.900
	95% CI	(0.705, 0.849)	(0.703, 0.877)	(0.70, 0.817)	(0.706, 0.863)
DL models	CNN	0.898	0.937	0.868	0.920
	LSTM	0.899	0.932	0.862	0.898
	RNN	0.888	0.971	0.856	0.911
	LN	0.910	0.966	0.853	0.914
	95% CI	(0.884, 0.913)	(0.92, 0.983)	(0.849, 0.87)	(0.896, 0.925)

### 3.2 The performance of DRFs, SEFs, and SRFs and their combinations

**Figure 5:**
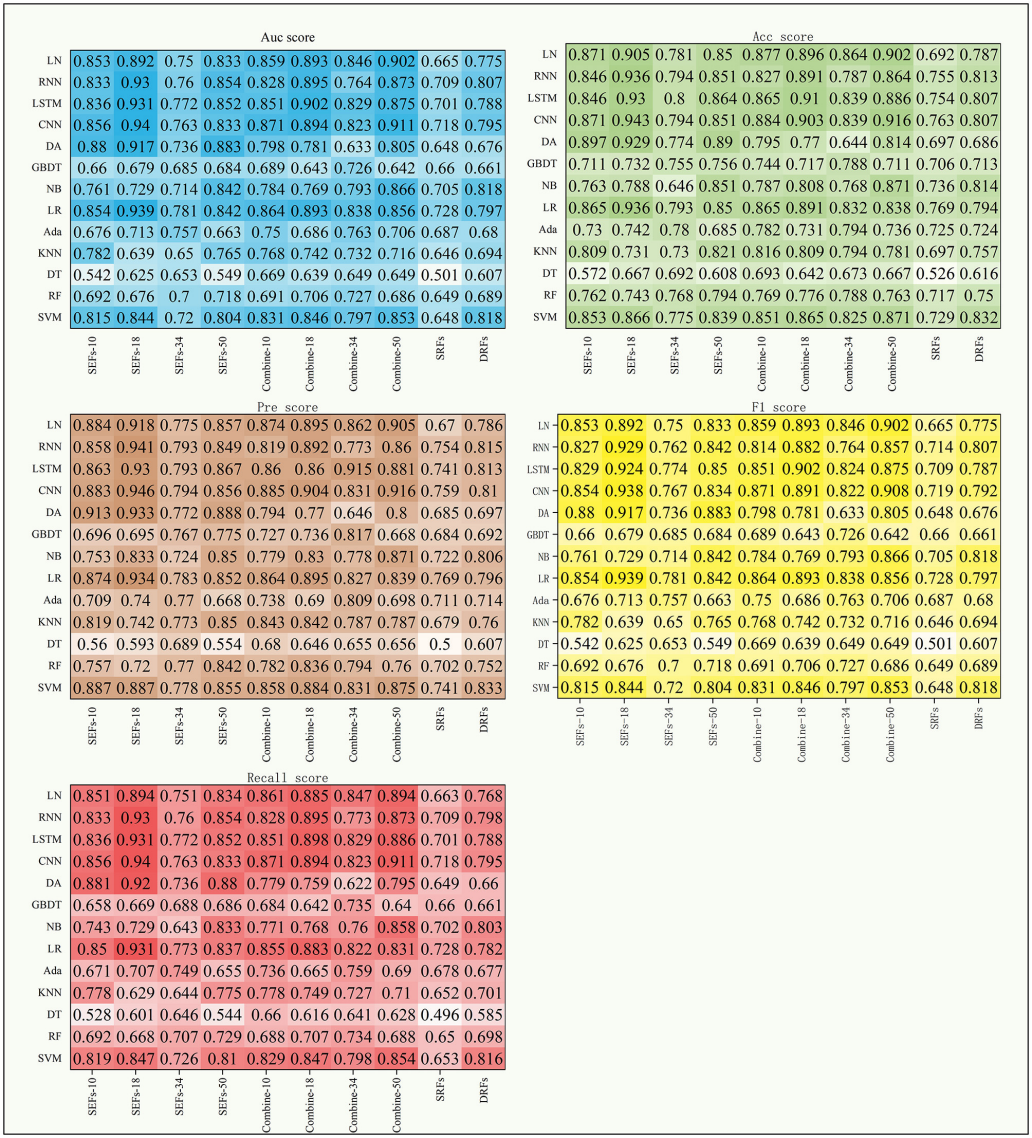
The scores of five indexes of SRFs, SEFs, and their combinations on the four DL models and nine ML models.

This study further concluded the performance of DRFs, SRFs, and SEFs extracted by the Lasso algorithm. As shown in Table 4 and Figure 5, except for SEFs-18 (the SEFs obtained from encoder Resnet 18), the best MS scores of the other five features on the DL models were lower than those on the ML models in general. The SEFs-18 achieved the best score of 0.936 in the ML model and 0.942 in the DL model. Besides, almost all four types of SEFs outperformed SRFs and DRFs. In detail, the SEFs-18 performed best among the four types of SEFs and achieved the best MS score of 0.936 on the ML model (LR) and 0.942 on the DL model (CNN), and the 95% CI were (0.678, 0.852) and (0.897, 0.955), respectively. SEFs-10 and SEFs-50 achieved an even result, wherein the SEFs achieved the best scores of 0.89 [95% CI (0.671, 0.835)] and 0.864 [95% CI (0.687, 0.845)] on the ML models and the DL models, respectively, while that of SEFs-50 was 0.885 [95% CI (0.687, 0.845)] and 0.857 [95% CI (0.836, 0.859)]. In addition, SEFs-34 achieved the same best score of 0.782 on the ML models and the DL models, and the 95% CI for both models were (0.696, 0.755) and (0.76, 0.787), respectively.

**Table 4:** The MS scores of DRFs, SEFs, and SRFs.

	**Models**	**SRFs**	**DRFs**	**SEFs-10**	**SEFs-18**	**SEFs-34**	**SEFs-50**
ML models	SVM	0.684	0.823	0.838	0.858	0.744	0.822
	RF	0.673	0.716	0.719	0.696	0.729	0.760
	DT	0.505	0.604	0.549	0.622	0.667	0.561
	KNN	0.664	0.721	0.794	0.676	0.689	0.795
	Ada	0.697	0.695	0.692	0.723	0.763	0.667
	LR	0.744	0.793	0.859	0.936	0.782	0.845
	NB	0.714	0.812	0.756	0.762	0.688	0.844
	GBDT	0.674	0.678	0.677	0.691	0.716	0.717
	DA	0.666	0.679	0.890	0.923	0.751	0.885
	95% CI	(0.618, 0.72)	(0.669, 0.78)	(0.671, 0.835)	(0.678, 0.852)	(0.696, 0.755)	(0.687, 0.845)
DL models	CNN	0.735	0.800	0.864	0.942	0.776	0.841
	LSTM	0.721	0.797	0.842	0.929	0.782	0.857
	RNN	0.728	0.808	0.839	0.933	0.774	0.850
	LN	0.671	0.778	0.862	0.900	0.762	0.842
	95% CI	(0.667, 0.761)	(0.776, 0.816)	(0.831, 0.873)	(0.897, 0.955)	(0.76, 0.787)	(0.836, 0.859)

Table 5 shows the MS scores of the four combined features. The DL models still achieved better results in this experiment. Among the four combined features, combine-50 including DRFs, SRFs, and SEFs (Resnet 50) and combine-18 including DRFs, SRFs, and SEFs-18 were the two best performers. The combine-50 achieved the best MS score of 0.866 on the ML model (NB) and 0.912 on the DL model (CNN), and the 95% CI for both models were (0.696, 0.827) and (0.857, 0.923), respectively. The combine-18 achieved the best MS score of 0.891 [95% CI (0.696, 0.823)] on the ML models and 0.897 [95% CI (0.889, 0.898)] on the DL models. Besides, the combine-10 including DRFs, SRFs, and SEFs-10 and combine-34 including DRFs, SRFs, and SEFs-34 obtained a similar score, where the best MS scores on the ML models were 0.862 (95% CI [0.722, 0.817)] and 0.831 [95% CI (0.7, 0.8)], and the best MS scores on the DL models were 0.876 [95% CI (0.819, 0.892)] and 0.853 [95% CI (0.767, 0.883)].

**Table 5:** The MS scores of combinations of DRFs, SEFs, and SRFs.

	**Models**	**Combine-10**	**Combine-18**	**Combine-34**	**Combine-50**
ML models	SVM	0.840	0.858	0.810	0.861
	RF	0.724	0.746	0.754	0.716
	DT	0.674	0.636	0.653	0.650
	KNN	0.794	0.777	0.754	0.742
	Ada	0.752	0.692	0.777	0.708
	LR	0.862	0.891	0.831	0.844
	NB	0.781	0.789	0.778	0.866
	GBDT	0.706	0.676	0.758	0.661
	DA	0.793	0.772	0.636	0.804
	95% CI	(0.722, 0.817)	(0.696, 0.823)	(0.7, 0.8)	(0.696, 0.827)
DL models	CNN	0.876	0.897	0.827	0.912
	LSTM	0.856	0.895	0.847	0.881
	RNN	0.823	0.891	0.772	0.866
	LN	0.866	0.892	0.853	0.901
	95% CI	(0.819, 0.892)	(0.889, 0.898)	(0.767, 0.883)	(0.857, 0.923)

## 4 Discussion

Previous studies (8–13) have used the image features of stroke lesions region and patients' basic information alone or in combination to predict patient stroke outcomes, resulting in excess time to segment stroke lesions and difficulty in improving accuracy. Some studies (29–31) have demonstrated the role of DRFs extracted from DSC-PWI images in predicting stroke outcomes. However, there is potential for improvement in stroke prediction accuracy based on a single whole brain DRFs. Considering that single DRFs may cause information loss, this study intended to process and extract whole brain images from other dimensions, aiming to obtain whole brain features of multidimensional and multi-state to save time for segmenting lesions and improve the accuracy of prognosis prediction. Based on the experimental results, the method proposed in this study successfully fused the whole brain dynamic and static features, and the combination of DRFs, SRFs, and SEFs achieved the best MS score of 0.971 both in machine learning models and deep learning models. The following four aspects discuss the methods used in this study, including the performance of DRFs, SRFs, SEFs, and their combinations, the effectiveness of the combined effect of whole brain dynamic and static features, the influence of different encoders on the prediction results, and the comparison with the results of the methods as mentioned above.

### 4.1 The performance of DRFs, SRFs, SEFs, and their combinations

The whole brain features extracted from different methods may provide different information and play their roles in predicting outcomes. This study concluded by comparing the performance of DRFs, SRFs, four types of SEFs, and their combinations. First, for the single DRFs, SRFs, and the four types of SEFs, the SEFs extracted from MinIP maps were more capable of predicting stroke outcomes (as shown in Table 4). The best MS scores of SEFs (Resnet-10), SEFs (Resnet-18), SEFs (Resnet-34), and SEFs (Resnet-50) were 0.89, 0.942, 0.782, and 0.885, respectively. All SEFs exhibited better scores compared to the best performance of SRFs (0.782) and DRFs (0.823). The SEFs and SRFs were extracted from the MinIP map, while DRFs were extracted from the PWI image. In contrast to the PWI image, which can directly characterize the dynamic blood flow of brain tissue, the MinIP map represents the lowest gray value of each voxel in the brain image when the contrast agent arrives, which can also be regarded as the static response of each voxel in the brain tissue. When the gray value is low, it indicates that the blood flow at this point is relatively rich and the blood volume is high. Thus, the transmission ability of brain tissue at the voxel can be reflected in the MinIP map, and the overall analysis of MinIP maps can express the static state of cerebral blood flow transmission and the state of brain injury. For the two static feature extraction methods, the Med3D model is a pre-trained network model, which can extract the global and local details of the MinIP map from the overall perspective, and the obtained SEFs can evaluate the whole brain tissue state relatively accurately. On the contrary, the SRFs extracted by radiomics are the overall analysis of the image's grayscale, texture, and shape. In terms of results, SEFs can provide more abundant prognostic information. In addition, dynamic blood flow information from DRFs may be more valuable than static information from SRFs. Although these extracted qualitative features fully represent the feature information of the brain image, it is not easy to establish a close correlation with the prognostic state due to the limitation of fixed features, and the prediction ability is lower than that of the whole brain coding features.

The direct combination of the three original whole brain features (combine-10, combine-18, combine-34, and combine-50) has a slight advantage over the single features. In detail, the best MS scores of combine-10, combine-18, combine-34, and combine-50 were 0.876, 0.897, 0.853, and 0.912, respectively, which were better compared to DRFs (0.823), SRFs (0.744), SEFs (Resnet-34) (0.782), but lower than that of SEFs (Resnet-18) (0.942). Thus, it can be concluded that the direct combination of primitive whole brain features failed to stably increase the predicting ability of the model, and even if it did, the improvement may not be large. Furthermore, this study further selected important features F_fuse_ from combined features in Table 5 and achieved a better score. Four groups of F_fuse_ achieved the best MS scores ranging from 0.868 to 0.971, which were better than the four types of combined features. Therefore, in the process of feature combination, it is necessary to perform feature screening, which is of great value to model optimization.

### 4.2 The performance of machine learning models and deep learning models

Since different models have their individual characteristics and suitable tasks, to fully verify the performance of the obtained features on different types of models, this study selected models from multiple perspectives of linear, non-linear, time characteristics, and feature dimensions for validation. The following models are commonly supervised ML models: SVM, DT, RF, LR, NB, and DA, while KNN is an unsupervised ML model. Besides, GBDT and Ada are the reinforcement ML models. For deep learning models, LN is the most traditional neural network model. Compared with CNN, RNN is more suitable for processing time series data based on its convolution characteristics and can achieve time memory and prevent the disappearance of the gradient. By adopting these 13 models, a reliable evaluation experiment can be conducted.

In general, the MS scores of the DL models were better compared to the ML models among the 13 models used in this study. Among the 9 ML models, the performance of LR was better compared to other models in most of the experiments. It achieved the best MS scores when input features were F_fuse_ based on Resnet 10, Resnet 18, and Resnet 34 (as shown in Table 3); SRFs, SEFs-18, and SEFs-34 (Table 4); and combine-10, combine-18, and combine-34 (Table 5). By introducing a non-linear sigmoid activation function, the LR model shows a significant advantage compared with single linear classification models. Among DL models, the CNN model achieved a better performance by not only exhibiting a better MS score than most of the ML models but also outperforming other DL models. Although RNN and LSTM provided the predictive ability of time dimension, they have not achieved effective results in this study. Therefore, it can be concluded that a model selection step is necessary when using different features as input for the model.

### 4.3 A comparison with the results of the previous methods

With the continuous innovation and development of medical technology, many stroke outcome prediction algorithms based on texture features (7, 10), multi-modal brain images (11, 14), and a combination of both (9) have been proposed. Since the text or image information is used directly in singular, the improvement of prediction accuracy is limited. Studies (30, 32) extracted DRFs from PWI images to supplement the dynamic information of blood flow and strengthen the characterization of the blood flow status of brain tissue. The results of these methods were compared and discussed here.

For the methods using patient basic information, this study collected the age, gender, NIHSS, onset time, lisp out, confusion, hypertension, diabetes, and atrial fibrillation as input data and utilized the model introduced in the study (7). The Auc score of 0.867 and the MS score of 0.831 were obtained. For the methods using DRFs to predict outcomes in studies (30, 32), the Auc scores of 0.828 and 0.934 were obtained. When using fusion strategies of focal and whole brain features, the best Auc score was 0.971, which is the same as our methods. It can be seen that the outcome prediction method based on the combined dynamic and static features of the whole brain proposed in this study can achieve the same score as the method based on the fusion features of the local and the whole brain. It means that the whole brain features from different methods may provide local information on ischemic lesions and supplement the features of local lesions. However, since the method used in our study does not require a quantitative analysis of the ischemic region, there is no need to perform the lesion segmentation process. In practical application, the lesion segmentation process is reduced, the efficiency of outcome prediction will be accelerated, and the clinical demand for rapid treatment and precision medicine for stroke can be better met.

### 4.4 The limitations and prospects of this study

The reliability of models presents a challenge as a larger dataset is needed to provide a better guarantee. Although the sample size of our study is relatively small which may lead to the vibration of experimental results, this study adopted several measures to reduce its influence. To reduce the confusion caused by the small sample size, we performed 10-fold cross-validation method to calculate five evaluation indexes (Auc, Acc, Pre, F1, and Recall) and obtained their MS scores on multiple models. Thus, a more reliable and equilibrium result can be obtained. Besides, we have also continued to collect new and external datasets to validate the methods presented in this study. Furthermore, we found that the performance of the different models varied widely. Some features performed better on one or more models, but when the features changed, the order of the models also changed. Therefore, we will carry out further work on the proposed stable performance model in the future.

Although the segmentation of stroke lesions was removed in our study, a skull step was needed to be performed. However, since the segmentation of stroke lesions needs to train models depending on a large number of datasets, it is more difficult and more time-consuming to accurately segment the stroke lesions. Moreover, inaccurate segmentation will seriously affect the results of feature analysis. In contrast, the skull step is easier to perform and consumes less time. Eventhough partial skull segmentation becomes wrong, it will not cause significant deviation in results due to a minor effect on the characteristics of the whole brain region.

Furthermore, since the size of the whole brain region is bigger than that of stroke lesions, it may need more time (several seconds) to extract features from the whole brain region than from stroke lesions. In general, the time difference only exists in the feature extraction stage. After determining the key feature F_fuse_, these features can be extracted directly in the practical application. In this case, the effect of time is negligible. Thus, even though multiple methods (Radiomics, Med 3D) were used for feature extraction in this study, it will not consume much time in practical application. Typically, at least 10 samples can be processed per minute.

Indeed, our method is proposed to predict the prognostic status of stroke patients, to assist clinicians in selecting more accurate treatment options and reducing the incidence of poor prognosis. However, before entering the clinic, we need to conduct further validation of the prediction model, such as adding multi-source external validation sets to verify the reliability of the model. This is one of our follow-up works.

## 5 Conclusion

This study proposed an outcome prediction method to improve the accuracy and efficacy of ischemic stroke outcome prediction based on the combined strategy of diverse whole brain features. From the experiments, the feature F_fuse_ generated from DRFs, SRFs, and SEFs (Resnet 18) achieved the best MS score of 0.971 both on machine learning models and deep learning models, and the 95% CI were (0.703, 0.877) and (0.92, 0.983), respectively. Besides, the deep learning models generally performed better than the machine learning models. The method used in our study can accurately assess stroke outcomes without segmenting ischemic lesions, which is of great significance for rapid, efficient, and accurate clinical stroke treatment.

## Data availability statement

The original contributions presented in the study are included in the article/supplementary material, further inquiries can be directed to the corresponding author.

## Ethics statement

The studies involving human participants were reviewed and approved by the Ethics Committee of Shanghai Fourth People's Hospital affiliated with the Tongji University School of Medicine, China (Approval Code: 20200066-01; Approval Date, 15 May 2020). The studies were conducted in accordance with the local legislation and institutional requirements. The participants provided their written informed consent to participate in this study.

## Author contributions

YY: Formal analysis, Methodology, Project administration, Resources, Supervision, Validation, Visualization, Writing – original draft, Writing – review & editing. YG: Conceptualization, Data curation, Investigation, Project administration, Software, Writing – original draft, Writing – review & editing.
